# Health-related quality of life in patients with dual diagnosis: clinical correlates

**DOI:** 10.1186/1477-7525-10-106

**Published:** 2012-09-05

**Authors:** Irina Benaiges, Gemma Prat, Ana Adan

**Affiliations:** 1 Department of Psychiatry and Clinical Psychobiology, University of Barcelona, Passeig de la Vall d’Hebron, 171, 08035, Barcelona, Spain; 2 Institute for Brain, Cognition and Behavior (IR3C), Barcelona, Spain

**Keywords:** Quality of life, Health-related quality of life, Dual diagnosis, Substance use, Severe mental illness

## Abstract

**Background:**

Although the studies published so far have found an affectation in the Health Related Quality of Life (HRQOL) in both psychiatric and substance use dependence disorders, very few studies have applied HRQOL as an assessment measure in patients suffering both comorbid conditions, or Dual Diagnosis. The aim of the current study was to assess HRQOL in a group of patients with Dual Diagnosis compared to two other non-comorbid groups and to determine what clinical factors are related to HRQOL.

**Methods:**

Cross-sectional assessment of three experimental groups was made through the Short Form – 36 Item Health Survey (SF-36). The sample consisted of a group with Dual Diagnosis (DD; N = 35), one with Severe Mental Illness alone (SMI; N = 35) and another one with Substance Use Dependence alone (SUD; N = 35). The sample was composed only by males. To assess the clinical correlates of SF-36 HRQOL, lineal regression analyses were carried out.

**Results:**

The DD group showed lower scores in most of the subscales, and in the mental health domain. The group with SUD showed in general a better state in the HRQOL while the group with SMI held an intermediate position with respect to the other two groups. Daily medication, suicidal attempts and daily number of coffees were significantly associated to HRQOL, especially in the DD group.

**Conclusions:**

The DD group showed lower self-reported mental health quality of life. Assessment of HRQOL in dual patients allows to identify specific needs in this population, and may help to establish therapeutic goals to improve interventions.

## Background

In the last two decades there has been an increasing interest in Quality of Life (QOL) and Health related Quality of life (HRQOL) as an assessment measure in care interventions, adverse effects of treatment and the impact of the illness through time [1], especially in psychiatric population [2]. Different studies have found an affectation both in the QOL and in the HRQOL in psychiatric disorders such as schizophrenia and bipolar disorder [3-7], as well as in substance dependence disorders [8-10].

Assessment of both QOL and HRQOL in Dual Diagnosis (DD) patients may help to identify areas of specific and clinical attention, given the special characteristics of this population: faster relapses [11], higher rates of rehospitalization and imprisonment [12], lower participation in the health services, more loss of social support and financial problems [12-14]. All these factors may be indicative of a lesser QOL in patients with DD, and its detection and clinical assistance could improve the efficacy of the interventions. Both QOL and HRQOL may represent useful measurements to assess the efficacy of such interventions.

Although there are few studies on the QOL in DD, most of the data published up to now show a worse QOL in these patients. Singh et al. [15] obtained a worse general QOL in DD patients with bipolar disorder compared with bipolar patients without comorbid Substance Use Dependence (SUD), with patients with SUD alone and with a normal control group. Kilbourne et al. [16] found that illicit drug use was associated with a decreased mental HRQOL in patients with bipolar disorder, and this effect continued one year later, even after controlling for the maniac and depressive symptoms of the disorder. Kalman et al. [17] obtained a lower mental HRQOL in a sample of dual patients with heterogeneous psychiatric disorders, compared with subjects with Severe Mental Illness (SMI) alone and with alcohol dependence alone. Bizarri et al. [18] also found a worse score in all the assessed domains of QOL in DD patients with opium dependence compared to patients without a concomitant mental disorder, and the differences were more marked in the domains of mental and physical functioning. In the study by Fassino et al. [19], the heroin dependent patients with comorbid personality disorder presented a worse QOL than those heroin dependent without the comorbidity.

However, some works do not obtain such differences, as for example in Astals et al. [20], which assessed the HRQOL in patients with and without a concomitant psychiatric disorder under treatment for heroin dependence. In the study by Garg et al. [21], the DD had worse scores in the dimension of Vitality but not in mental and physical health domains. Finally, Wade et al. [22], in a sample of young patients with a first psychotic episode, observed that the affectation in both QOL and social functioning were related to the level of severity in substance use. Thus, the QOL in patients with mild consumption did not differ from that of non-consumers, while it was worse in patients with heavy consumption. The variety of definitions on QOL, sample characteristics, design of the studies and different instruments and procedures applied can be explanatory factors of the heterogeneity of results.

The aim of our study was to assess HRQOL through SF–36 in a group of patients with DD, and compare it to the group with SMI and to the third group with SUD as well as to establish comparisons to published values for the normal Spanish population according to the mean age of our sample and male gender norms [23]. To our knowledge, no other previous research has worked with this design, including these three experimental groups and their comparisons to normative data. We expected higher scoring in the SUD group, indicative of a better HRQOL state, while the SMI group would be in the medium scoring range and DD would present the worse HRQOL scores, especially in the mental health domain. In an exploratory way, we further sought to determine which clinical factors would be related to HRQOL, with special interest in the DD group.

## Material and methods

### Sample and procedure

This study is part of a larger study on health related quality of life, neurocognitive functioning and personality traits in patients with DD. In the present paper, we only present the data concerning health-related quality of life.

In a prospective cross-sectional design we enrolled 125 male patients aged 18 to 60 years, divided in three groups. Two groups with a severe mental illness (schizophrenia, bipolar disorder, or major depressive disorder) with comorbid SUD (DD) and without comorbid SUD (SMI), and they were consecutively admitted to “social club program” at the Mental Health Division of the Althaia Foundation in Manresa (Barcelona, Spain). A third group of patients with SUD without psychiatric comorbidity (SUD) was under treatment on the therapeutic community of the Gressol Catalonia Man Project (Barcelona), between January 2010 and June 2011. Each participant was consecutively referred by their treating psychiatrist, who was blind to the aims of the study, and had been diagnosed using the Structural Clinical Interview for DSM-IV-R [24] Axis I Disorders (SCID-I) [25]. The inclusion criteria were: (1) Current diagnosis of schizophrenia, bipolar disorder or major depression; or current diagnosis of substance use dependence in remission for at least four months and absence of relapses at least one month before the study participation; (2) male gender; (3) age between 18–60 years. The SUD group has the additional criterion of no other current DSM-IV-R criteria for any Axis I or II disorders. The exclusion criteria were: no DSM-IV-R criteria for a current substance-induced psychiatric disorder or psychiatric disorder due to a medical condition, unstable or severe medical illness, mental retardation, history of traumatic brain injury or neurological injury, violent behavior and having received electroconvulsive therapy within 12 months prior to their study participation.

**Figure 1:**
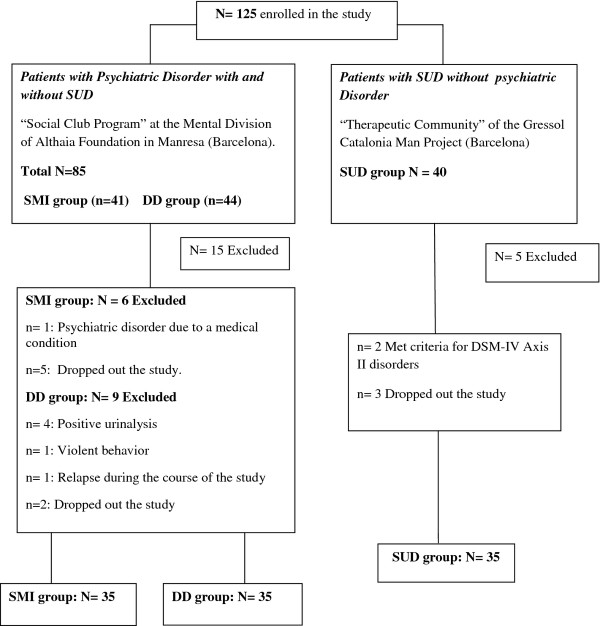
**Participants flow-chart.** DD, Dual Diagnosis; SMI, Severe Mental Illness; SUD, Substance Use Dependence.

The procedure of the study was divided into 4 one hour long sessions. The data presented in this study was collected in the first session by a doctoral level psychologist, and written informed consent was obtained from all participants after the procedures of the study were fully explained to them. In the following sessions we carried out neurocognitive and personality assessments. A urine drug screen was performed between the first and the second session of the study. After this initial assessment and during the course of the study, 20 patients were excluded. The analyses were performed with a total sample of 105, divided into 35 subjects in each group (see Figure 1).

This study was approved by the ethics committees of the University of Barcelona and the Mental Health Division of Althaia, meeting the ethical principles of the Declaration of Helsinki.

### Instruments

#### ***Clinical measures***

Information was collected by means of a *structured interview* of sociodemographic (age, marital status, social class, schooling and economic status) and clinical variables (diagnosis, psychiatric and substance use family history, age of onset of the disorder and/or consumption, relapses, abstinence periods, type of drug used, suicidal attempts, presence of organic pathology and medication). We reviewed the medical records of the patients, contrasting the self-reported data with the medical history on the database of the hospital and with their treating psychiatrist. Daily consumption of cigarettes and cups of coffee as well as other intakes of beverage with caffeine, such as tea or cola, were recorded. Smokers were administered the *Fagerström* test [26] of nicotine dependence. Additionally, the *Clinical Global Impression*[27] was applied as a subjective measure of the clinical severity of each participant.

#### ***Health- related quality of life***

To assess the HRQOL we selected the SF-36 questionnaire [28] (Short Form −36 Item Health Survey), one of the most used and adequate generic instruments in psychiatric [29,30] and drug addicted population [31]. The Spanish version has good psychometric properties and reference population values [23]. The SF-36 provides scores in 8 dimensions: Physical Functioning, Role - Physical, Role - Emotional, Social Functioning, Mental Health, General Health, Bodily Pain, and Vitality, with scores ranging from 0 to 100, where a higher score indicates a better functioning in the scale. It also has an additional item measuring the Health Changes perceived by the subject in comparison to the previous year (Health Transition item). The questionnaire provides two composite standardized scales in T scores with mean 50 and standard deviation 10: the Physical health Component Summary (PCS) and the Mental health Component Summary (MCS). These two scales explain 80 to 85% of the variance in the 8 original dimensions and have shown greater reliability [32].

#### ***Statistical analysis***

We calculated the descriptive statistics for all the variables. The possible intergroup differences (DD, SMI and SUD) in the sociodemographic and clinical variables were explored using univariate (ANOVA) and multivariate analyses (MANOVA) for the continuous data. Categorical data were examined with Chi Square tests in the case of variables with nominal scales (marital status, economic situation, living situation, type of medical comorbidities, and type of psychotropic medication, psychiatric diagnosis, type of intake of substance/dependence and degree of dependency on the Fagerström tests).

The intergroup differences in the HRQOL were examined by MANOVAs and later introduced covariates in analysis (MANCOVA) to explore the variance of its effect on the HRQOL. In all cases, we estimated the power sample so that the statistical test would not reject the null hypothesis wrongly by not detecting an effect if there were one. We considered values from 0.70 as suitable. The statistic partial squared Eta (η_p_^2^) was also estimated to measure the effect size, that is, the degree to which each factor is affecting the dependent variable, where a value of 0.01 was low, 0.04 moderate and 0.1 high [33]. Both statistics are used to avoid the occurrence of type II error. All analyses were Bonferroni corrected due to the multiple comparisons carried out in order to control the possibility that the statistical tests would find an effect where there was not one. Thus, this correction method prevents from the occurrence of Type I error.

Finally, in an exploratory way, we carried out a bivariate correlational analysis between the demographic and clinical variables with the summatory scores of the PCS and the MCS. We intended to identify the variables of interest to be introduced in the following lineal regression analysis, trying to find clinical correlates of the HRQOL. The analyses were done with the statistical package SPSS (version 15.0) and the tests were considered bilaterally with a type I error established at 5%.

## Results

### Sociodemographic and clinical variables

Table 1 and Table 2 summarize the sociodemographic and clinical data of the sample. Groups were equivalent in age, number of children, years of schooling and type of cohabitation. There were intergroup differences in marital status (*χ*^2^ = 9.891; p = 0.007), with a higher percentage of singles in the SMI than in both DD and SUD groups, and in the economic status (*χ*^2^ = 26.651; p < 0.0001), with a higher percentage of subjects receiving a disability pension in both DD and SMI groups compared to a higher percentage of active workers in SUD group (See Table 1).

**Table 1:** Sociodemographic data for the three groups of patients

**Sociodemographics**	**DD (N = 35)**	**SMI (N = 35)**	**SUD (N = 35)**
Age	37.91 ± 8.34 yr	38.63 ± 8.73 yr	36.37 ± 6.72 yr
Marital status			
Single	60.0%	85.7%	51.4%
Stable partner	11.4%	0%	8.6%
Married	2.9%	5.7%	11.4%
Separate/Divorced	11.5%	8.6%	28.5%
Widower	2.9%		
Number of children	0.49 ± 1.09	0.17 ± 0.51	0.51 ± 0.78
Living			
Alone	20.0%	8.6%	2.9%
Accompanied	77.1%	91.4%	88.6%
Years of study	9.14 ± 2.01	9.66 ± 2.44	9.58 ± 2.23
Economic situation			
Active	8.6%	14.3%	42.9%
Disability pension	65.7%	74.3%	17.1%
Unemployed	11.4%		20.0%
No income	8.6%	11.4%	20.0%

*DD*, Dual Diagnosis; *SMI*, Severe Mental Illness; *SUD*, Substance Use Dependence.

**Table 2:** Clinical data for the three groups of patients

**Clinical data**	**DD (N = 35)**	**SMI (N = 35)**	**SUD (N = 35)**
Psychiatric Diagnosis (% = 100)			
Schizophrenia	45.7%	80%	
Bipolar Disorder	28.6%	14.3%	
Major Depression Disorder	25.7%	5.7%	
Age of psychiatric disorder onset	24.82 ± 7.47 yr	25.67 ± 8.07 yr	
Mean duration of illness (yr)	12.27 ± 7.81 yr	14.09 ± 8.66 yr	
Number of relatives with SUD	0.35 ± 0.69	0.06 ± 0.23	0.23 ± 0.59
Number of relatives with psychiatric disorder	0.65 ± 1.01	0.86 ± 0.87	0.49 ± 0.74
Number of suicidal attempts	1.60 ± 3.2	0.34 ± 0.68	0.34 ± 0.87
Number of medical comorbidities ^a^	0.66 ± 1.05	0.57 ± 0.73	0.46 ± 0.78
Asthma/Allergy	11.4%	8.6%	2.9%
Triglycerides/Cholesterol	11.4%	25.7%	2.9%
Diabetes	2.9%	2.9%	2.9%
Obesity		2.9%	
Hypertension	2.9%	8.6%	2.9%
HIV	5.7%		14.3%
Hepatitis B or C	5.7%	2.9%	5.7%
Others	14.3%	2.9%	5.7%
Daily number of medications^a^	3.86 ± 1.83	3.03 ± 1.20	0.69 ± 1.10
Typical antipsychotics	20.0%	17.1%	2.9%
Atypical antipsychotics	57.0%%	60.0%	5.7%
Antidepressants	28.6%	25.7%	11.4%
Mood stabilizers	25.0%	31.4%	8.6%
Anxiolytics	40%	22.9%	8.6%
Other medication	31.4%	25.7%	20%
Clinical Global Impression (CGI)	4.69 ± 0.96	4.50 ± 0.74	3.26 ± 1.01
Daily number of cigarettes	18.34 ± 12.41	10.43 ± 12.44	14.74 ± 8.08
Fagerström score	5.11 ± 2.52	2.89 ± 3.38	3.86 ± 2.48
No dependence	14.3%	54.3%	17.1%
Low dependence	8.6%		20.0%
Moderate dependence	45.7%	25.7%	51.4%
High dependence	31.4%	20%	11.4%
Daily Number of coffees (cups)	1.77 ± 1.73	1.17 ± 1.44	1.66 ± 1.43
Other daily beverages with caffeine	0.86 ± 1.00	0.71 ± 1.04	0.91 ± 1.26

*DD*, Dual Diagnosis; *SMI*, Severe Mental Illness; *SUD*, Substance Use Dependence.

^a^ Percentage will not equal 100 as each participant may suffer from more than one medical illness or take more than one medication.

With respect to the clinical variables (Table 2), we obtained main effects in number of daily medications (F_(2,102)_ = 47.162; p = 0.0001; η_p_^2^ = 0.048). The DD group was taking more daily medications than the SMI group (p = 0.016) and the latter more than the SUD group (p = 0.0001). Considering the type of psychotropic medication, both DD and SMI groups were taking more typical and atypical antipsychotics, mood stabilizers, anxiolytics and than the SUD group (*χ*^2^ ≥ 8.864; p ≥ 0.012).

The DD group also had more suicidal attempts (F_(2,102)_ = 4.630; p = 0.010; η_p_^2^ = 0.083) than the SMI and SUD (p = 0.010) groups, without difference between the latter two groups.

A higher score in the Fagerström test was found in the DD and SUD groups (F _(2,102)_ = 5.453; p = 0.006; η_p_^2^ = 0.097) with higher daily consumption of cigarettes (F_(2,102)_ = 4.405; p = 0.015; η_p_^2^ = 0.080). Although the groups did not differ in number of medical comorbidities, the analysis by type of medical illness showed a higher prevalence of HIV infection in the SUD group (*χ*^2^ = 5.816; p = 0.050) and an increased incidence of triglycerides and cholesterol in the SMI group compared with the other two (*χ*^2^ = 7.467; p = 0.024). The CGI showed a worse clinical state in both DD and SMI groups (F_(2,102)_ = 25.097; p = 0.0001; η_p_^2^ = 0.033) compared to the SUD group (p = 0.0001). We did not find any differences in family psychiatric history, family SUD history and daily number of coffees or other beverages containing caffeine. No differences emerged between the DD and SMI groups in type of psychiatric diagnosis, age of onset of mental illness and years of illness duration.

Table 3 summarizes the data on substance consumption for both DD and SUD groups. They did not differ in number or type of intake, number of substances used, type of substances used, months of abstinence, number of relapses, age of onset of substance use or duration of SUD disorder.

**Table 3:** Substance Use data in Dual Diagnosis (DD) and Substance Use Dependence (SUD) patients

**Substance use data**	**DD (N = 35)**	**SUD (N = 35)**
Type of intake		
One substance	25.7%	20%
Two substances	25.9%	22.9%
Polydrug use	48.4%	57.1%
Number of substances used	2.80 ± 1.79	3.14 ± 1.53
Substance Abuse/Dependence^a^		
Cocaine	100%	100%
Cannabis	54.3%	48.6%
Alcohol	71.4%	68.6%
Ecstasy	28.6%	25.7%
Hallucinogens	17.1%	14.3%
Opioids	22.9%	28.6%
Sedatives	5.7%	2.9%
Months of abstinence	13.41 ± 14.31	9.14 ± 5.07
Number of relapses	1.35 ± 2.52	0.54 ± 1.03
Age of intake onset (yr)	19.82 ± 7.32	20.00 ± 8.27
Mean duration of SUD (yr)	16.85 ± 7.32	15.60 ± 7.16

^a^ Percentages will not equal 100 as each participant may take more than one substance of abuse.

### HRQOL comparisons

The results obtained in the 8 subscales and the two composite scales of the SF-36 are shown in Table 4, as well as the contrasts among groups. The analyses provided main significant differences in most of the subscales, except for Bodily Pain. The Health Transition item and the composite scale MCS have also proved significant.

**Table 4:** Mean and standard deviation in the SF-36 and results of the MANOVAs

				**MANOVA**			
**SF-36**	**DD (N = 35)**	**SMI (N = 35)**	**SUD (N = 35)**	**F**	**Effect size**	**Power sample**	**Contrasts**^**a**^
Subscales							
Physical Functioning	86.40 ± 14.32	93.67 ± 9.23	96.00 ± 8. 20	7.06**	0.12	0.92	DD < SMI, SUD
Role- Physical	70.31 ± 39.36	92.64 ± 24.25	76.71 ± 40.58	3.50*	0.06	0.64	DD < SMI/DD = SUD
Role-Emotional	46.87 ± 44.69	83.32 ± 34.09	68.55 ± 39.57	7.04**	0.12	0.92	DD < SMI/DD = SUD
Social Functioning	60.56 ± 28.77	82.05 ± 26.98	80.71 ± 24.87	6.58**	0.11	0.90	DD < SMI, SUD
Mental Health	50.12 ± 19.27	62.47 ± 18.89	65.60 ± 17.22	6.47**	0.11	0.89	DD < SMI, SUD
General Health	50.46 ± 20.69	57.47 ± 21.07	73.74 ± 14.18	13.60**	0.21	0.99	DD, SMI < SUD
Bodily Pain	67.65 ± 25.24	77.32 ± 27.35	72.34 ± 25.95	1.12	0.02	0.24	
Vitality	42.19 ± 16.36	51.91 ± 20.59	68.00 ± 16.54	17.76**	0.26	1.00	DD, SMI < SUD
Health Transition Item	63.28 ± 31.09	63.97 ± 24.76	92.14 ± 20.80	14.03**	0.22	0.99	DD, SMI < SUD
Composite Scales							
PCS	50.31 ± 9.18	52.82 ±7.11	53.66 ± 7.80	1.54	0.03	0.32	
MCS	34.61 ± 13.02	44.03 ± 12.49	45.42 ± 10.63	7.82**	0.13	0.94	DD < SMI, SUD

*DD*, Dual Diagnosis; *MCS*, Mental health Component Summary; *PCS*, Physical health Component Summary; *SMI*, Several Mental Illness; *SUD*, Substance Use Dependence.

^a^ We detail only the significant contrasts.

* p < 0.05; ** p < 0.001.

The post-hoc analysis revealed a worse score for the DD group in most of the subscales except in Role-Physical and Role-Emotional compared to the SUD group. The DD also obtained worse scoring in the Health Transition item and in the MCS scale compared to the SUD group. The DD group also scored lower than the SMI group in most of the HRQOL domains except for General Health and the Health Transition item. The SUD and SMI groups only differed in Vitality, General Health and in the Health Transition item, without differences in the rest of the scales (see Table 4).

In view of significant group differences on certain clinical variables, especially relevant in the DD group, we carried out the analyses again introducing number of daily medication, suicidal attempts and number of daily cigarettes as covariates. The significance was maintained for the scales of Physical Functioning (F_(2,102)_ = 4.924; p = 0.009; η_p_^2^ = 0.082), Role - Emotional (F_(2,102)_ = 4.592; p = 0.012; η_p_^2^ = 0.088), Social Functioning (F_(2,102)_ = 3.012; p = 0.050; η_p_^2^ = 0.060), Vitality (F_(2,102)_ = 9.818; p = 0.0001; η_p_^2^ = 0.171) and the Health Transition item (F_(2,102)_ = 8.556; p = 0.0001; η_p_^2^ = 0.153) with power sample values of ≥ 0.760 in all cases. There were again no differences in the subscale of Bodily Pain and the PCS scale. Previous main differences in Role - Physical, Mental Health, General Health subscales and in the MCS disappeared when the covariates were introduced.

The contrast *a posteriori* only maintained the differences between the DD and SUD groups in Physical Functioning (p = 0.031), Vitality (p = 0.0001) and in the Health Transition item (p = 0.004), with a better score in the SUD group. This group also presented a better score in Vitality (p = 0.008) and in the Health Transition item (p = 0.0001) with respect to the SMI group. The differences between the DD and SMI groups were maintained for Physical Functioning (p = 0.015), Role - Emotional (p = 0.016) and Social Functioning (p = 0.050), with lower scores in the DD group.

**Figure 2:**
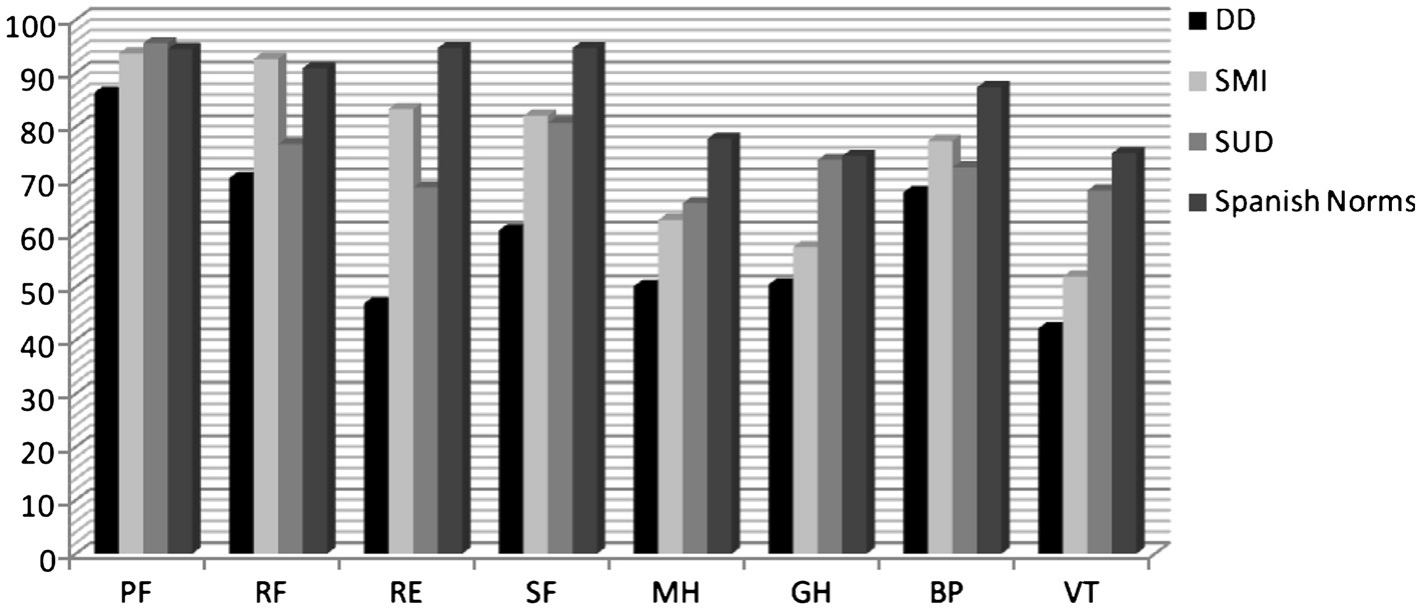
**Mean scores for the three groups of patients and the Spanish norms in the 8 scales of the SF-36. **PF, Physical Functioning; RF, Role – Physical; RE, Role – Emotional; SF, Social Functioning; MH, Mental Health; GH, General Health, BP, Bodily Pain; VT, Vitality.DD, Dual Diagnosis; SMI, Severe Mental illness, SUD, Substance Use Dependence.

**Figure 3:**
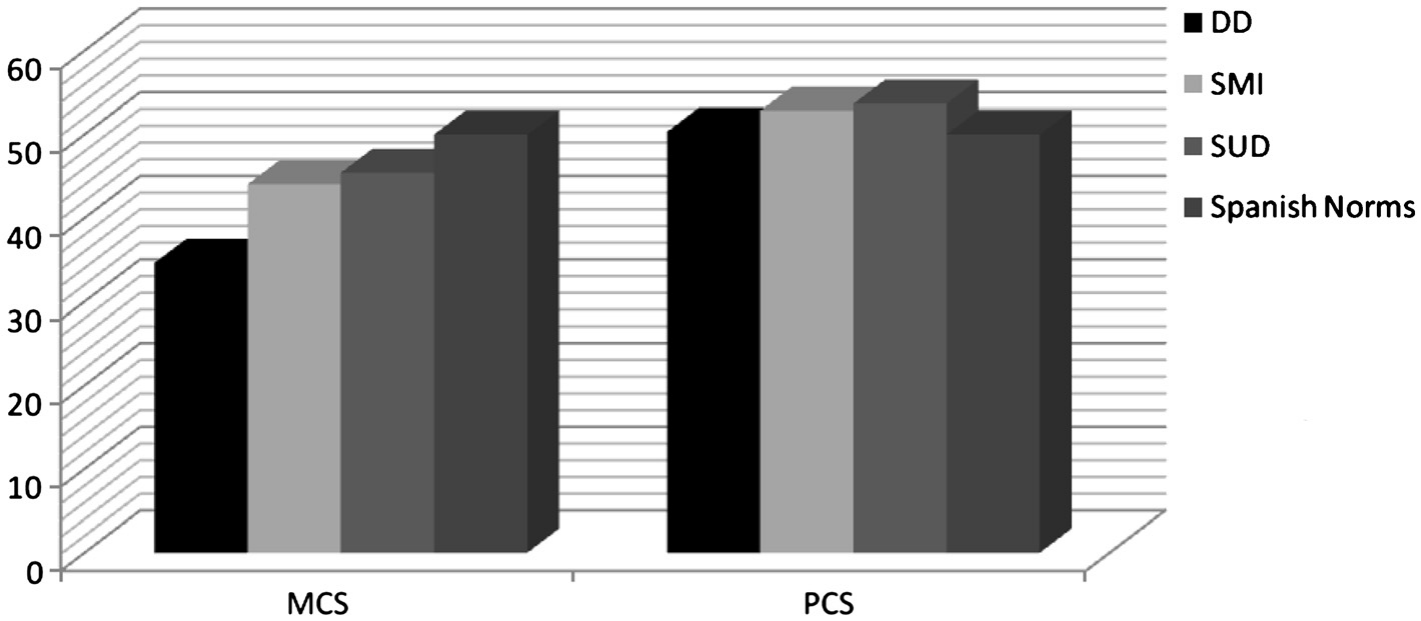
**Mean scores for the three groups of patients and the Spanish norms in the MCS and the PCS. **MCS, Mental Health Component Summary; PCS, Physical Component Summary. DD, Dual Diagnosis; SMI, Severe Mental Illness, SUD, Substance Use Dependence.

The comparison of the group means with the Spanish normative data [23] showed that, although all the groups generally presented lower scores, this was more marked in the DD group. The DD scores in the scales of Social Functioning, Role - Emotional, Mental Health, General Health and Vitality were below the norm. The SMI group presented one standard deviation below the norm only in the Vitality scale and the SUD group did not present scores below the norm in any scale (see Figure 2). Regarding the composite scales, the DD group was the only one that was one standard deviation below the norm in the MCS scale (see Figure 3).

### Factors contributing to HRQOL

The correlational analysis in the whole sample did not provide any significant association between the score in the PCS scales and the sociodemographic and clinical variables. In contrast, the score in the MCS scale showed negative associations with the number of suicidal attempts (r = −0.268; p = 0.007), medication (r = −0.303; p = 0.002) and daily number of coffees (r = −0.209; p = 0.036). The correlational analysis taking into account each group of patients only provided a significant association in the DD group between the MCS scale and the daily number of coffees (r = −0.497; p = 0.004). The suicidal attempts and the medication did not reach significance in any group.

These three variables were later introduced as independent variables in a lineal regression analysis with the whole sample and the MCS scale as a dependent variable. The analysis of the general model was significant (F = _(3,102)_ = 6.536; p = 0.0001), explaining 14.2% of the variance in the MCS scale. The results showed that daily number of medications was related to the mental health component of the HRQOL (see Table 5). When the same analysis was done for each group, the general model only maintained the significance in the DD group (F_(3,32)_ = 4.224; p = 0.010), explaining 21.8% of the variance, where the only significant variable was daily consumption of coffee (see Table 5).

**Table 5:** Lineal regression for the MCS for the total sample and for the Dual Diagnosis group

**MCS**	**Adjusted R**^**2**^	**IV**	**ß standardized**	**p values**	**Tolerance**	**VIF**
TS (N =105)	0.142	Coffee^ª^	−0.179	0.059	0.975	1.025
		Medication ^b^	−0.265	0.006	0.960	1.042
		Suicidal Attempts	−0.188	0.052	0.937	1.067
DD (N = 35)	0.218	Coffee^ª^	−0.468	0.007	0.975	1.026
		Medication ^b^	−0.171	0.290	0.997	1.003
		Suicidal Attempts	−0.134	0.413	0.997	1.023

*DD*, Dual Diagnosis; *IV*, Independent Variables; *MCS*, Mental Health Component Summary; *TS*, Total sample; *VIF*, Variance Inflation Factor.

^ª^Daily number of coffees.

^b^Daily number of medications.

## Discussion

As we expected, the DD group presented worse HRQOL scores in most of the scales compared to the other two groups, especially in the mental health domain assessed by the MCS scale. In contrast, the groups did not differ in the scale of Bodily Pain or in Physical functioning (PCS). In general, the SUD group had better scores in most of the scales of the SF-36, with marked differences with respect to the DD group, while the SMI group obtained intermediate scores.

The introduction of the covariates suicidal attempts, medication and daily consumption of cigarettes as variables that may be potentially associated to the HRQOL and QOL [34-36] kept the worst HRQOL scores in the DD group except for Mental Health, General Health and MCS, where the three groups had similar scores. After the adjustment, the SUD group showed a better feeling of vitality and energy, and a better perception of the evolution of the health state with respect to the previous year (Health Transition item), compared to the SMI and DD groups. The SUD group also showed a better perception of the physical health state compared to the DD, while there were no differences in the rest of the scales. These data suggest that the SUD group shows a lower functional disability, and this could be related to a better community functioning, as suggested by the fact that in this group there is a higher percentage of subjects who are active workers.

Thus, suicide attempts, number of daily medication and daily cigarette consumption appear to modulate the differences between the DD and SUD groups regarding HRQOL expression. These variables should be taken into account in further research, since they may be determinant in DD scores on scales assessing the mental domain of HRQOL. However, after the adjustment on these clinical variables, the DD group continued showing a lower degree of physical and emotional health, with higher perceptions of limitations in daily social and occupational life, as well as a higher feeling of tiredness and exhaustion compared with the SMI group. This result, in agreement with the work by Kalman et al. [17], may highlight the moderating negative effect of substance use on the relationship between psychiatric disorder and mental HRQOL in patients with DD.

This is also in line with the work by Kilbourne et al. [16], who observed a negative association between substance consumption and the mental domain of the HRQOL in bipolar patients, even after controlling for the depressive and manic symptoms of the disorder. Our results suggest a major detrimental effect on HRQOL in people with a psychiatric disorder with comorbid SUD than in those without comorbid SUD or other psychiatric disorders, despite clinical severity and other variables related to substance use, such as type of substance used or time of abstinence. Although our results agree with several previous studies [15-19], most of them only compared DD and SUD patients, and they were lacking the specific impact of the mental disorder related to HRQOL in DD patients.

Regarding factors associated to HRQOL, the MCS scale was negatively related with the number of suicidal attempts, daily number of medication and daily number of coffees. When there were more suicidal attempts, more daily medication intake and/or a higher daily number of coffees, the score in the MCS scale was lower. These variables were related to a worse state in the mental domain of the HRQOL. However, the separate analysis by groups showed that the model was met only in the case of DD patients, and that caffeine consumption was the best predictive variable. Although this result indicated that in DD patients the high number of coffees taken is related to a higher affectation in the mental domain of the HRQOL, coffee consumption in this group was not higher than in the SMI or SUD groups.

Caffeine consumption, as well as other methylxanthines, has long been associated with psychiatric illness, possibly due to its beneficial impact on extrapyramidal side effects of antipsychotic medication through dopaminergic agonism in the nigrostratial pathways [37]. However, caffeine consumption may have detrimental effects on DD patients through dopaminergic agonists in other brain pathways such as the mesolimbic and the mesocortical, resulting in an increase of psychotic symptomatology. Since intraindividual variations have been described in the psychoactive effects of caffeine in the general population [38], our results point to the possibility that the DD population is the most sensitive one to the effects of caffeine, even under moderate doses compared to the other two groups.

Different hypotheses could explain this result. One explanation could be that the DD group may present different genetic polymorphisms from the general population, both in metabolic enzymes and in brain receptors to the effects of caffeine. A more plausible explanation may be that caffeine covaries with other risk factors associated with the DD group: more suicidal attempts, higher daily medication intake, and higher cigarette consumption. Thus, caffeine intake has been associated with higher cigarette consumption [39], and the consumption of both substances has been in turn associated with a higher risk of suicidal attempts in the psychiatric population [40,41] and to a lower efficacy of the pharmacological treatment [39,40]. All these factors, at the same time, have been directly or indirectly associated with a worse QOL and HRQOL [34-36,42,43].

Further research is needed in this area, exploring the HRQOL state in the DD population, taking into account the consumption of nicotine and caffeine in order to explore their impact on HRQOL and with which risk factors their effects may be associated. Likewise, studying the QOL/HRQOL in DD women is of great interest, since there is evidence of a worse state in the mental domain in female substance users [21,44]. Thus, given the differences in HRQOL state between sexes, it should be noted that our results may be representative only of male DD patients.

### Strengths and limitations of the study

In our study, we bring improvements over previous research on this topic. The group comparisons to the Spanish normative data have provided evidence of the degree of impairment in the assessed domains of HRQOL. We also bring new data on the major factors associated with DD and lower mental HRQOL such as suicidal attempts, cigarette smoking, daily caffeine intake and number of prescribed medications. All these clinical factors are rarely taken into account in previous studies. Likewise, the control of abstinence time by urinalysis and the calculations of power sample in all analyses are important methodological factors preventing the influence of possible intermediate variables and their control has provided strength to our results.

However, the limitations of the study should also be noted. Although the cross-sectional study design allowed us to ascertain the weight of each psychiatric condition in HRQOL of DD, it did not allow us to investigate causal relationships between substance disorders, psychiatric comorbidities and HRQOL. We failed to analyze the independent effects of different types of substances on HRQOL because a vast percentage of subjects in our sample were polyconsumers. Another important limitation is that the study was based on clinically acquired and partially retrospective self-reported data that may have been subject to recall bias, including information about suicidal acts and substance intake. The heterogeneity of the psychiatric disorders included and the relatively small sample size might have affected the representativeness of the sample. Further, the inclusion of only males makes difficult to interpret our findings as specific characteristics of the subjects suffering from DD since the results cannot be extrapolated to female gender. Noteworthy, we did not include the information of the subjects who dropped out the study or were excluded, so it is unknown whether the results are subject to bias due to the exclusion of these subjects. Finally, all subjects in our sample were contemplating treatment maybe affecting the generalisability of the results to those subjects who were not considering treatment.

## Conclusions

Overall, our findings show a worse state in the mental domain of the HRQOL in the DD patients with respect to the other two groups without comorbidity and to the general population. Suicidal attempts, a higher intake of daily medication and caffeine consumption appear as factors associated to the impairment in the mental domain of the HRQOL, especially in the DD group. The systematic assessment of the HRQOL in DD patients should allow us to improve our knowledge of its associated factors. It may also be a useful tool in the detection of areas of specific assistance to the goals of treatment planning, thereby improving the effectiveness of the intervention and the assessment of the results.

## Abbreviations

BP: Bodily Pain; CGI: Clinical Global Impression; DD: Dual Diagnosis; GH: General Health; HT item: Health Transition on Time item; MCS: Mental health Component Summary; MH: Mental health; PCS: Physical Health Component Summary; PF: Physical Functioning; RE: Role-Emotional; RF: Role-Physical; SF: Social Functioning; SF-36: Short-Form 36 Item Health Survey; SMI: Severe Mental Illness; SUD: Substance Use Dependence; VT: Vitality.

## Competing interests

The authors declare that they have no competing interests.

## Authors' contributions

AA conceived the original idea for the study, sought funding, wrote the protocol and managed the day to day running of the study. IB collected the data of the sample and carried out all data analyses. The manuscript was written by IB and AA with input from GP. All authors have approved the final manuscript.
